# A Comparison of Individual Learning and Social Learning in Zebrafish Through an Ethorobotics Approach

**DOI:** 10.3389/frobt.2019.00071

**Published:** 2019-08-14

**Authors:** Yanpeng Yang, Romain J. G. Clément, Stefano Ghirlanda, Maurizio Porfiri

**Affiliations:** ^1^Key Laboratory of Mechanism Theory and Equipment Design of Ministry of Education, School of Mechanical Engineering, Tianjin University, Tianjin, China; ^2^Department of Mechanical and Aerospace Engineering, New York University, Tandon School of Engineering, Brooklyn, NY, United States; ^3^Department of Psychology, Brooklyn College, Brooklyn, NY, United States; ^4^Departments of Psychology and Biology, The Graduate Center of the City University of New York (CUNY), New York, NY, United States; ^5^Centre for the Study of Cultural Evolution, Stockholm University, Stockholm, Sweden; ^6^Department of Biomedical Engineering, New York University, Tandon School of Engineering, Brooklyn, NY, United States

**Keywords:** behavior, biomimetics, ethorobotics, observational learning, robotics

## Abstract

Social learning is ubiquitous across the animal kingdom, where animals learn from group members about predators, foraging strategies, and so on. Despite its prevalence and adaptive benefits, our understanding of social learning is far from complete. Here, we study observational learning in zebrafish, a popular animal model in neuroscience. Toward fine control of experimental variables and high consistency across trials, we developed a novel robotics-based experimental test paradigm, in which a robotic replica demonstrated to live subjects the correct door to join a group of conspecifics. We performed two experimental conditions. In the individual training condition, subjects learned the correct door without the replica. In the social training condition, subjects observed the replica approaching both the incorrect door, to no effect, and the correct door, which would open after spending enough time close to it. During these observations, subjects could not actively follow the replica. Zebrafish increased their preference for the correct door over the course of 20 training sessions, but we failed to identify evidence of social learning, whereby we did not register significant differences in performance between the individual and social training conditions. These results suggest that zebrafish may not be able to learn a route by observation, although more research comparing robots to live demonstrators is needed to substantiate this claim.

## 1. Introduction

Social learning is widespread among animals, contributing significantly to behavioral adaptation in both individuals and groups (Zentall and Galef, 1988; Leadbeater and Chittka, 2007; van Schaik, 2010; Hoppitt and Laland, 2013). In addition to elucidating a crucial adaptive mechanism, studies of animal social learning can lead to improved understanding of human pathologies to which social learning contributes, such as anxiety and phobias (Blanchard et al., 2001; Delgado et al., 2006; Mineka and Zinbarg, 2006), or in which it is affected, such as in autism spectrum disorders (Schneider and Przewłocki, 2005; Markram et al., 2008).

A long-standing question is whether social learning can be explained by associative learning mechanisms or whether it requires more sophisticated learning abilities (Heyes, 2012b; Lind et al., 2019). Within this debate, observational learning is of special interest. Observational learning refers to learning a behavior from simple observation, without the opportunity for practice. In standard associative learning theory, learning an action requires performing it (instrumental conditioning; Pearce, 2008; Bouton, 2016). Hence, evidence of observational learning of actions would indicate a learning mechanism that is more sophisticated than associative learning (Lind et al., 2019), or possibly a modified associative mechanism (Heyes, 2001, 2012a).

Here, we study observational action learning in zebrafish, *Danio rerio* (Engeszer et al., 2007). The use of zebrafish in developmental biology has produced in-depth knowledge and powerful tools for genetic experimentation (Vascotto et al., 1997), which is being leveraged in behavioral genetics and neuroscience (Norton and Bally-Cuif, 2010). Genetic similarities with mammals (Crollius and Weissenbach, 2005) have also established zebrafish as a prime model organism for translational clinical research (Stewart et al., 2012). However, the potential of zebrafish in behavioral science is not fully realized because of the relative paucity of behavioral screening tools (Sison and Gerlai, 2010), and this is especially true in the case of learning (Gerlai, 2011). Our study is simultaneously an investigation of social learning and a contribution to the wider landscape of behavioral methods in zebrafish.

Social learning is common in fish (Brown and Laland, 2003), but existing studies do not conclusively establish learning of actions by observation. For example, fish can learn a route by following conspecifics (Laland and Williams, 1997; Laland and Williams, 1998; Reebs, 2000), but this allows them to practice the route and could be based on innate following behavior (Brown and Laland, 2003) in combination with associative learning (Lind et al., 2019). Anthouard (1987) demonstrated that naïve *Dicentrarchus labrax* learned an action more quickly after observing experienced conspecifics, but the setup enabled naïve fish to make partial responses, such as approaching and snapping, which may have facilitated learning. Because these are likely genetically predisposed responses to the sight of foraging fish (Brown and Laland, 2003), the study does not unequivocally support observational learning of an action.

Further evidence of observational learning come from studies with guppies (*Poecilia reticulata*) and sailfin mollies (*Poecilia latipinna*), showing that females can learn preferences for males by observing other females (Dugatkin and Godin, 1992, 1993; Schlupp and Ryan, 1997; Witte and Ueding, 2003; Godin et al., 2005). These results may derive either from observational action learning (learning to swim toward a specific male) or from observational learning of a preference for a stimulus (a specific male) coupled with a pre-existing response (swimming toward males in general). Because these studies bear some conceptual similarity to ours, we will consider them in more detail in the Discussion. Similarly, males *Astatotilapia burtoni* have been shown to infer the fighting ability of conspecifics by observations (Grosenick et al., 2007), but evidence that fish are capable of observational learning of actions remains scarce.

Robotics often take inspiration from nature (Brambilla et al., 2013; Kim et al., 2013; Valentini et al., 2016), but robots are also increasingly used to study animals. In order to advance our understanding of observational learning in fish, we established a novel ethorobotics-based experimental paradigm that could afford finer control of experimental conditions. Ethorobotics represent a promising interdisciplinary research area at the interface of ethology and robotics (Webb, 2000; Partan et al., 2009; Krause et al., 2011; Halloy et al., 2013; Frohnwieser et al., 2016; Porfiri, 2018; Romano et al., 2018), in which robots whose design is inspired by animals help understanding animal behavior by allowing fine-tuned interactions. Our paradigm uses a robotic zebrafish replica as a demonstrator in order to control precisely what information is displayed to the subject. The replica is built to mimic the morphology, size, coloration, and motion of live zebrafish. Its motion is controlled in two dimensions (2D) via a Cartesian plotter, which allows for the implementation of realistic swimming patterns, in terms of both movement trajectory and body undulations. In previous work, we showed that equivalent robotic replicas elicit approach responses in live fish, similar to social behavior that is generally exhibited toward conspecifics (Ruberto et al., 2016, 2017; Kim et al., 2018). For example, zebrafish show a similar preference for associating with a replica and a conspecific in binary choice tests (Ruberto et al., 2017).

Here, to ensure that learning could proceed only by observation, rather than by practicing the correct behavior, we confined subjects in a small area during demonstrations. The task consisted of learning to approach one of two doors in order to gain proximity to a shoal of conspecifics. Subjects in the social training condition observed the robotic replica approaching both the incorrect door, to no effect, and the correct door, whose opening is triggered automatically by a real-time video tracking system. Subjects in the individual training condition learned without the demonstrator and provided a control group. In this task, zebrafish learned a preference for swimming to the correct door, but we observed no effect of social vs. individual training: fish that observed the demonstrator did not learn more quickly, and did not spend more time in proximity of the correct door compared to fish who learned individually. We consider the implications of these results and further developments in the Discussion.

## 2. Materials and Methods

This section is organized as follows. First, we detail the robotics-based experimental setup, focusing on both the hardware and software. Then, we present the experimental procedure, including the animals, the structure of the trials, and the experimental groups used in the study. Finally, we articulate our data analysis, consisting of a wide range of behavioral and learning measures, along with multivariate statistical models. All data and code for analysis are available as Supplementary Information.

### 2.1. Robotics-Based Experimental Setup

#### 2.1.1. Hardware

**Figure 1:**
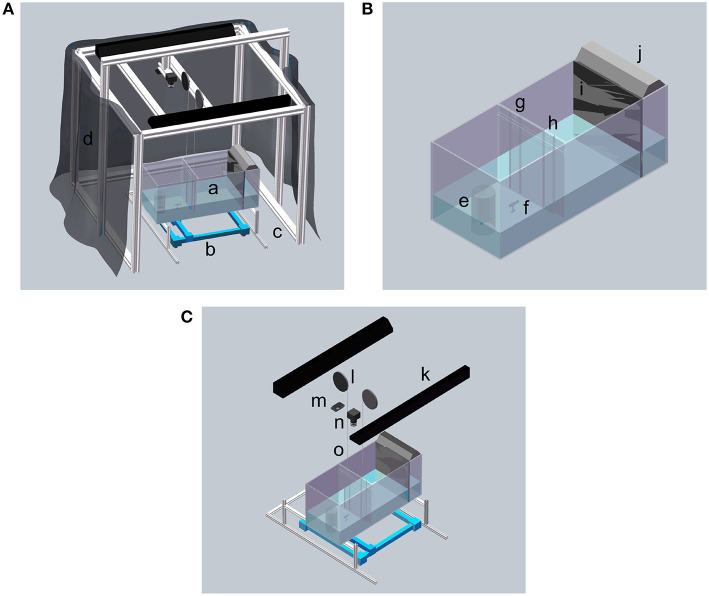
Schematic of the experimental apparatus. **(A)** Overview of the experimental apparatus. **(B)** Detailed view of the experiment tank. **(C)** Detailed view of the hardware. a Experimental tank; b 2D Cartesian plotter; c Aluminum frame made by T-slot bars; d Curtain; e Transparent cylinder and live zebrafish; f Zebrafish replica; g and h Acrylic transparent doors; i One-way glass; j 12-inch light; k 36-inch light; l Pulley system; m Logitech webcam; n Flea3 camera; and o Fishing line.

The experiment was performed in a glass tank (74 × 30 × 30 cm; length, width, and depth) supported by a custom frame built with T-slot bars (McMaster, Robbinsville, NJ, USA), shown in Figure 1A. The bottom of the tank was raised 29 cm above floor level to fit the Cartesian plotter used to maneuver the replica. To minimize extraneous visual stimuli, dark curtains were mounted around the tank. The bottom and side walls of the tank were covered by white contact paper (McMaster, Robbinsville, NJ, USA) to ease video tracking.

The tank was divided into three sections with lengths of 30, 34, and 10 cm using two partitions: a transparent partition with two doors and a one-way glass partition, see Figure 1B. The one-way glass partition, with thickness of 5.9 mm, was used to house a shoal of 10 zebrafish, preventing them to see the subject and interact with it. The lateral section delimited by the partition with the doors is the focal compartment where subject behavior was monitored. The middle section was intended to maintain some distance between the subject and the stimulus group, such that the subject would need to explore the partition with the doors to gain proximity to the group.

The doors were cut from a transparent acrylic sheet (McMaster, Robbinsville, NJ, USA), and they were held in place by acrilic guides glued to the main partition, so that they could only move along the vertical direction. Each door was 1.5 body lengths (BLs) wide to allow the subject and the replica to smoothly transit through them. The doors were located at 1/4 and 3/4 of the width of the partition, symmetrically with respect to the middle horizontal axis.

Each door was connected to a pulley via a transparent fishing line (Berkley Trilene XT Extra Tough, Pure Fishing, Inc., Columbia, SC, USA), shown in Figure 1C. The pulleys (external diameter of 13 cm and internal diameter of 12 cm) consisted of a 3D printed plastic plate and a servo motor (HS-5086 WP, Hitec RCD USA, Inc., Poway, CA, USA). The motors were activated by a microcontroller (Arduino Uno, Arduino Srl, Italy).

**Figure 2:**
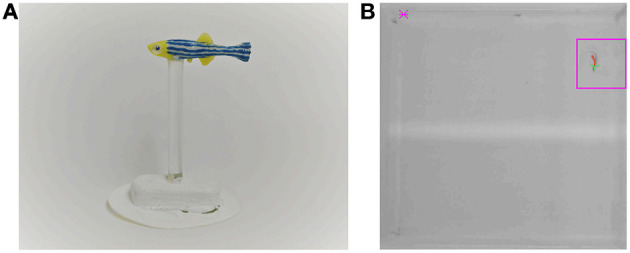
Zebrafish replica and tracking system. **(A)** Zebrafish replica used to study social learning. **(B)** A screen shot of the tracking system. The magenta square represents the monitored region in front of one of the door. The door would open upon detection of the subject within this region.

The replica was fabricated using a 3D-printed mold (Ultimaker 2+, Ultimaker B.V., Geldermalsen, The Netherlands), where we poured a flexible silicone mixture (Smooth-On, Inc., Macungie, PA, USA), see Figure 2. The use of silicone instead of rigid material allows a more naturalistic bending of the replica's body during its motion through the experimental tank, which could increase its biomimicry and acceptance by the live zebrafish (Romano et al., 2017, 2019a). The replica was then painted with silicone-based paint (Smooth-On, Inc., Macungie, PA,USA). The replica was attached to a transparent rod, clamped to a 3D-printed base, which, in turn, was magnetically connected to a Cartesian plotter (XY Plotter Robot Kit, Makeblock Co., Ltd, Shenzhen, China) to control its motion. The plotter was placed below the tank to minimize acoustic and visual confounds. As discussed in a separate, focused publication, this platform enables realistic swimming motion of the robotic replica with accurate positioning and fast reaction time (DeLellis et al., submitted).

Above the tank, we installed two cameras at a height of 137 cm from the floor, see Figure 1C. A Logitech C920 (Newark, CA, USA) webcam was used for tracking the position of the subject in the focal compartment with a resolution of 640 × 480 pixels. A Flea3 FL3-U3-13E4C USB camera (FLIR Integrated Imaging Solutions Inc., Richmond, BC, Canada), with a higher resolution of 1280 × 1024 pixels was used to capture the entire experimental tank and monitor the subject's interaction with the shoal, for reward timing. This camera was controlled by software FlyCapture SDK (FLIR Integrated Imaging Solutions Inc., Richmond, BC, Canada).

Uniform illumination was provided by two 36-inch, 30 W white fluorescent lights (All-Glass Aquarium Co., Inc., Franklin, WI, USA) mounted along the sides of the tank at a distance of 110 cm from the floor. A third light, a 12-inch fluorescent strip light with a power of 8 W (All-Glass Aquarium Co., Inc, Franklin, Wisconsin, USA), was used for additional illumination of the stimulus region so that the group could be seen clearly by the subject, see Figure 1B.

#### 2.1.2. Software

The apparatus was operated from a PC using a custom software developed in Matlab 2018a (The MathWorks, Inc., Natick, MA, USA). Live tracking of the subject fish was based on Matlab computer vision toolbox, including detection of moving objects and localization of object centroids. At each tracking step, two gray-scale frames were acquired by the Logitech C920 Pro camera and clipped to a fixed region of interest containing the tank. Frames were captured at 20 Hz. The first frame was subtracted from the second, yielding an image with outlines of the fish and, if present, of the replica. This image was processed to remove noise, fill in the outlines of the targets, and estimate the targets' positions from the centroids of the filled outlines. The fish and replica were distinguished from each other by using the input to the Cartesian plotter. If this procedure failed to locate the fish, a Kalman filter was used to extrapolate from previous frames; in DeLellis et al. (submitted), details of the tracking system are presented.

The tracking system also monitored a square region of 2 × 2 BL in front of the correct door to detect the presence of the subject fish, see Figure 2. The latter could be opened and closed by sending appropriate commands from the PC to an Arduino Uno controller. The replica was controlled by programming sequences of 2D coordinates and sending them from the PC to another Arduino microcontroller. The sequence was generated by implementing a stochastic mathematical model of zebrafish swimming, which we have established in our previous work (Mwaffo et al., 2015, 2017; Zienkiewicz et al., 2018). The model captures the typical burst-and-coast swimming style of zebrafish, where sudden tail beats are followed by longer coasting phases. Details of the implementation are presented in DeLellis et al. (submitted).

### 2.2. Experimental Procedure

#### 2.2.1. Animals

Zebrafish were purchased from Carolina Biological Supply Co. (Burlington, NC, USA). We used a total of 56 fish: 36 fish were used as focal subjects, with a female/male ratio of 5:4 and an average BL of around 3 cm. An equal number of focal subjects (18) were used for each condition. The remaining 20 fish were used to form the stimulus shoals, with an equal sex ratio and similar average BL as the experimental subjects.

Animals were housed in 37.5 L (10 gallons) vivarium tanks (Pentair Aquatic Eco-systems Locations, Cary, NC, USA) with a density of no more than 10 fish per tank. Water temperature and acidity were kept at 26° and 7.2 pH. Housing lights were maintained for a period of 12 h light/12 h dark. The fish were fed commercial flake food (Nutrafin max; Hagen Corp., Mansfield, MA, USA) once per day around 7 PM.

After the fish habituated to the housing tank for at least 15 days, they were individually tagged with silicone-based visible implant elastomers (VIEs) (Northwest Marine Technology Inc., Shaw Island, WA, USA). Before tagging, the colored part and the curing agent of the VIE were mixed with a proportion of 10:1, and the fish was anesthetized to avoid unnecessary wounds. The VIE was injected bilaterally on two locations near the head. Tag colors were randomly selected and combined among white, purple, blue, and yellow. After tagging, the fish were given at least 14 days of recovery in their housing tank.

#### 2.2.2. Trial Structure

The experiment investigated whether zebrafish would learn to open a door in order to join a shoal of 10 conspecifics, visible behind a one-way glass, see Figure 1. Each subject was trained for 20 trials either individually, where it would learn alone how to open the correct door, or socially, where it could observe a robotic zebrafish replica demonstrate door opening at the beginning of each trial.

**Figure 3:**
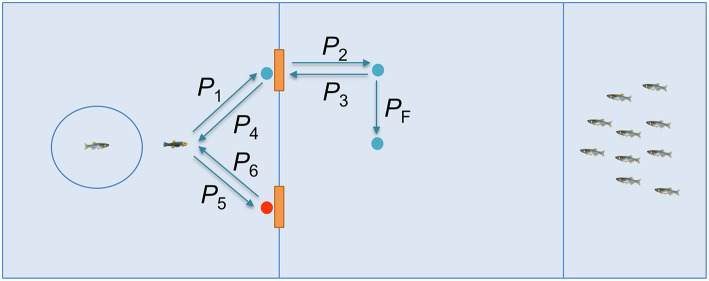
Illustration of the demonstration by the replica in the social training condition.

For the first 10 min of each trial, experimental subjects were confined in the focal region via a transparent plastic cylinder (diameter 8 cm). During this time, subjects in the social training condition observed the robotic replica demonstrate door opening as sketched in Figure 3, while subjects in the individual training condition simply waited.

The demonstration by the replica entailed the following steps. At the beginning of each trial, the replica interacted with the focal subject for 30 s, following a trajectory generated via our stochastic model of zebrafish locomotion (Mwaffo et al., 2015, 2017; Zienkiewicz et al., 2018) with an attraction point at the center of the cylinder that housed the experimental subject. This resulted in the replica swimming in the focal region, while frequently approaching the subject and “wall kissing” the cylinder. The replica then swam in a straight line to the correct door (*P*_1_) and started tail beating for 3 s. As a result, the door would open and the replica swam through (*P*_2_), before stopping for 5 s while beating its tail. After these 5 s, the replica would go back to the focal region (*P*_3_ and *P*_4_) to resume the interaction with the subject for another 30 s. Then, it approached the wrong door and station there, beating its tail for 3 s, but the door would not open. After this cycle was repeated 6 times over 10 min, the robotic replica transited through the correct door and move to the final position (*P*_F_), facing the stimulus group until the subject finished the task.

At the end of the first 10 min of each trial, subjects were released from the cylinder and allowed to swim freely in the focal compartment until they opened the door, within a time limit of 30 min. The open door allowed subjects to access to the central compartment, bringing focal subjects closer to the shoal of conspecifics. The door would open if the focal subject stationed in a 6 × 6 cm, unmarked zone in front of the correct door (Figure 2) for at least 3 s out of any 5 s. The triggering process was controlled automatically through the tracking system described above. Once the door opened, the subject were rewarded by being allowed to swim for 2 min close to the conspecifics in the central compartment.

Learning was assessed by measuring changes in proximity to the two doors across learning trials, as well as during three 30 min tests conducted before the first trial, after trial 10, and after trial 20. During these three additional tests, both doors remained closed, and no robotic replica was present. The subject was confined in region A for 10 min and then released in region B for an additional 10 min.

Correct functioning of the apparatus was tested prior to the beginning the experiment, using several pilot fish not included in the experiment. Sample videos of individual and social training are provided as Supplementary Information. Some of these trials along with a preliminary description of the experiment have been presented in a recent meeting (Yang et al., 2019).

Upon inspecting the data, we discovered that performance was consistently better when subjects had to swim to one of the doors, and that this preferred door changed between the first two batches, that is, depending on the orientation of the apparatus. This pattern indicates the presence of an uncontrolled factor external to the apparatus, which biased exploration toward one of the two sides. We have thus coded all data to indicate whether the correct door was, for each subject, on the overall preferred or non-preferred side.

We discovered this bias after completing the individual training condition, and we kept the same experimental layout for the social training condition to ensure that the data were comparable. We speculate that fish might have been attracted to the familiar sound of the housing tanks, which were ~2 m from the tank on the preferred side. In the future, we will orient the apparatus so that the housing tanks will lie behind the shoal of conspecifics, thus reinforcing their attractive effect rather than introducing a side bias.

#### 2.2.3. Experimental Groups

The experiment ran from June to September 2018. In each trial, only one fish was trained. Each fish (a total number of 36) was tested twice per day, once in a morning session between 9 a.m. and 1 p.m., and once in an afternoon session between 2 and 6 p.m. Each condition (individual or social training) was performed on two batches of nine subjects each. The assignment of the correct door was fully counterbalanced across conditions, batches, and subjects. A consistent sex ratio of five females to four males was used in each batch. In between trials, subjects were housed in four tanks, keeping together individuals of the same sex that were assigned the same correct door. Twenty more fish were used as stimuli, split into two groups of 10 individuals each. In both conditions, the stimulus group used in the morning sessions of the first batch was used in the afternoon sessions of the second batch, and vice-versa.

Before each training session, the tank was filled with new tap water and a drop of coating (AcquaSafe Plus, Tetra, Blacksburg, VA, USA) to neutralize pollutants, such as chlorine, chloramines, and heavy metals, and strengthen bacterial beds. The water height was always 10 cm and the temperature was maintained at around 27° C.

### 2.3. Data Analysis

#### 2.3.1. Behavioral and Learning Measures

The raw data collected in the experiments consisted of the subject's trajectory and the door triggering time acquired by the real-time tracking system. From these data, we computed the parameters defined in Table 1 to measure behavior and learning. The two main measures of learning are *T*, the time between the release of the subject from the cylinder and the door opening (right-censored at 30 min on unsuccessful trials) and preference index (PI), defined as the time spent in proximity of the correct door over the time spent in proximity of either door (that is, within the region used to trigger door opening, see Figure 2). If learning is successful, we expect *T* to decrease over trials and PI to increase from a value close to 0.5 (no preference) to a value above 0.5 (preference for the correct door).

**Table 1:** Behavioral and learning measures.

**Symbol**	**Description**	**Formula**
*T*	Time between subject release and door opening	—
τ_*C*_	Time spent in a 6 × 6 cm region in front of the correct door	—
τ_*I*_	Time spent in a 6 × 6 cm region in front of the incorrect door	—
*T*_*i*_	Time spent within 3 cm of wall *i*	—
PI	Preference index for the correct door	τ_*C*_/(τ_*C*_ + τ_*I*_)
RI	Reward index for associating with conspecifics (reward value)	$T_{1}/\overset{4}{\underset{i=1}{\sum }}T_{i}$
*H*	Entropy of the trajectory	$-\overset{100}{\underset{i=1}{\sum }}P_{i}\log_{2}P_{i}$
θ_*C*_	Angle between the fish heading and the direction to the correct door	
*v*_*t*_	Speed	$\frac{∥{\text{x}}_{t+1}-{\text{x}}_{t}∥}{\Delta_{t}}$
*a*_*t*_	Acceleration	$\frac{∥{\text{v}}_{t+1}-{\text{v}}_{t}∥}{\Delta_{t}}$
ω_*t*_	Turn rate	$\frac{1}{\Delta_{t}}\cos^{-1}\left(\frac{v_{t+1}\cdot \;v_{t}}{\left\|v_{t+1}\right\|\left\|v_{t}\right\|}\right)$
*F*	Freezing time (time that the fish moved <4 cm over a rolling window of 2 s)	—
*A*	Avoidance response for the door after it opened	—
PI_m_	Modified preference index for the replica, held in place at the middle of the tank width	—

*In the Formula column, — indicates a primary variable derived directly from video tracking. The walls are ordered such that wall 1 is the transparent partition separating regions A and B. In the formula for H, we partitioned the 30 × 30 cm region B into a 10 × 10 square grid, so that the length of each square is ~1 body length; therein, P_i_ is the fraction of video frames in which the subject was in grid cell i. Δ_t_ is the interval between frames, that is, 0.05 s. For calculations, raw trajectory data were smoothed using a moving average with a span of 4 frames, such that **x**_t_ is the smoothed 2D position in the tank*.

To fully characterize zebrafish behavior, we also computed the following measures:

- *H*, the entropy of the trajectory. If the subject learns to approach and open the door efficiently, its swimming should become less random and thus the entropy of the trajectory should decrease.
- θ_*C*_, correct heading, defined as the absolute value of the angle between the current heading of the fish and the vector from the fish to the center of the correct door. A value of 0° indicates swimming directly toward the door, while nonzero values indicate less precise swimming. This parameter and the following ones were computed based on recorded swimming trajectories.
- RI, the reward index that quantifies the subject's preference for swimming close to conspecifics vs. far from them. We used this measure to evaluate whether the stimulus shoal was attractive to the subjects, as assumed in our experimental setup.
- Locomotor activities, in terms of freezing time, average speed, average angular speed, and average acceleration (Macrì et al., 2017). We used these indices to evaluate whether exposure to the replica altered the behavior of the animals.
- *A*, avoidance response for the door after it opened, scored by evaluating whether the distance between the focal fish and the center of the correct doors reached a value larger than two body lengths within the 15 s following door opening. This parameter was computed using the first and last training trial for each fish, for a total of 72 videos overall.
- PI_m_, the modified preference index was used to measure the preference of focal fish for the replica, which was stationed between the two doors. We divided the tank into three rectangular regions of equal area along its width, and we computed this index as the fraction of time spent in the middle region of the tank.

#### 2.3.2. Statistical Model

Using linear mixed effects modeling, with subject as a random factor, we related door triggering time (*T*), preference for the correct door (PI), and the other variables in Table 1 to the independent variables “condition” (individual or social training), “correct door location,” and either “trial,” for data collected during training trials, or “test,” for data collected during test trials. These independent variables and their possible values are summarized in Table 2. The independent variable “correct door location” encapsulates the experimental bias that we observed in our data. Although the correct door was counterbalanced across subjects and the apparatus was rotated 180° in between the two batches of each condition, fish consistently displayed better performance when they had to swim to one of the two doors.

**Table 2:** Independent variables used in data analysis. See the text for details.

**Variable**	**Type**	**Values**
Condition	Unordered factor	Individual, Social
Test	Ordered factor	0, 10, 20
Training	Numeric	1, …, 20
Correct door location	Unordered factor	Preferred side, Non-preferred side

At first, a linear mixed full model with the global ID of the fish as random effect was built. Non-significant interaction terms were then discarded from the model. In order to correct for false positive due to multiple testing, we took into account that each independent variable entered two statistical tests relative to test data (preference index and heading), and three tests relative to training data (preference index, heading, and door triggering time). Conservatively, we applied an alpha level of 0.050/3 ≃ 0.017.

We also used Levene's test to investigate differences in variability of the dependent variables across different combinations of independent variables.

Data analysis was conducted in Emacs Org-mode (Dominik, 2010; Schulte et al., 2012) and R version 3.5.0 (R Core Team, 2018) with packages car (Fox and Weisberg, 2011), data.table (Dowle and Srinivasan, 2018), readxl (Wickham and Bryan, 2018), effects (Fox and Weisberg, 2018), and ascii (Hajage, 2009).

## 3. Results

### 3.1. Test Data

Test trials provide the best assessment of differences between social and individual training because they took place on days in which no training occurred and because, contrary to training trials, the replica was absent even in the social condition. Thus, any difference between conditions would be attributable to learning rather than to short-term influence of the replica, such as on emotional response.

**Figure 4:**
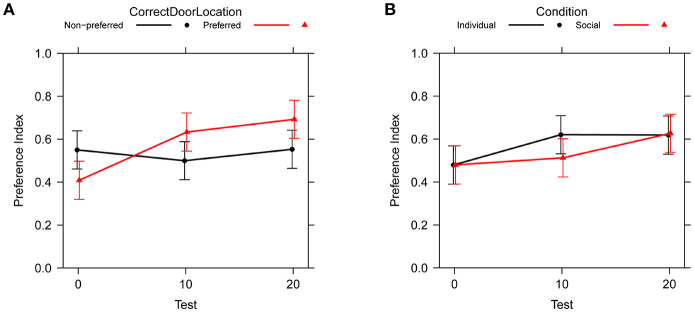
Change in preference index across test trials (PI in Table 1). Bars are 95% confidence intervals. **(A)** Comparison between preferred and non-preferred correct door location. **(B)** Comparison between social and individual training.

Table 3 shows type II ANOVA results for the preference between correct and incorrect door (PI in Table 1). We found a main effect of test, showing an improvement from no preference to about 62% preference for the correct door, and an interaction between test and location of the correct door, illustrating that the improvement over tests occurs primarily when the correct door is on the preferred side of the tank, see Figure 4. There was no effect of social vs. individual training, see Figure 4. There was also no significant difference between the variability of the preference across groups of subjects [Levene's test: *F*_(11, 96)_ = 1.13, *P* = 0.347].

**Table 3:** Type II ANOVA table for the preference index (PI) during test trials, as a function of training condition, correct door location, and test.

	**χ**^**2**^	**Df**	**p**
Condition	0.82	1	0.364
CorrectDoorLocation	1.44	1	0.230
Test	10.44	2	**0.005**
Condition:CorrectDoorLocation	0.91	1	0.339
Condition:Test	2.10	2	0.350
CorrectDoorLocation:Test	12.89	2	**0.002**
Condition:CorrectDoorLocation:Test	1.56	2	0.459

*Here and in the remaining tables, we write “CorrectDoorLocation” to identify with a single word the corresponding independent variable. Bold values indicate statistically significant results*.

**Figure 5:**
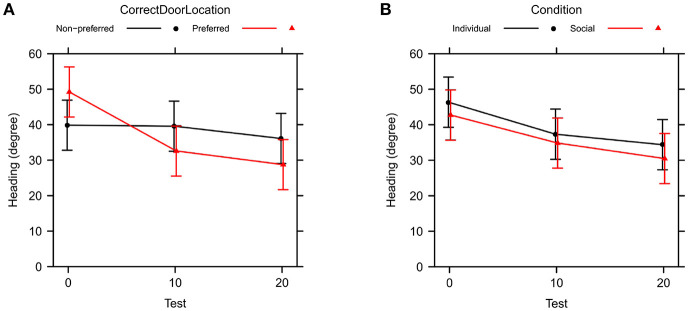
Change in heading precision toward the correct door across test trials (θ_*C*_ in Table 1). Bars are 95% confidence intervals. **(A)** Comparison between preferred and non-preferred correct door location. **(B)** Comparison between social and individual training.

A type II ANOVA of heading direction toward the correct door (θ_*C*_) yields similar results, see Table 4 and Figure 5. In addition, we found a significant interaction between training condition and correct door location, indicating less accurate heading for the social training condition when the correct door was on the preferred side of the tank, but not when the door was on the non-preferred side.

**Table 4:** Type II ANOVA table of heading direction during test trials, as a function of training condition, correct door location, and test.

	**χ**^**2**^	**Df**	**p**
Condition	1.55	1	0.213
CorrectDoorLocation	0.39	1	0.535
Test	14.30	2	**0.001**
Condition:CorrectDoorLocation	8.02	1	**0.005**
Condition:Test	0.05	2	0.975
CorrectDoorLocation:Test	8.45	2	**0.015**
Condition:CorrectDoorLocation:Test	0.07	2	0.964

*Bold values indicate statistically significant results*.

Approaching conspecifics appeared to be an adequate motivation for the focal fish, as they spent considerable time close to the wall with the doors. Of all the time spent within one body length (3 cm) of the walls, an average of 87% was spent near this wall.

The modified preference index, assessing the preference of the fish toward the replica, tended to decrease with the number of tests and did not show a significant variation between social and individual training. Examining the effect of the number of training sessions, we found that the tendency to explore the doors increased after 10 training trials (Supplementary Information).

We further investigated whether other behavioral variables differed across tests and conditions. Type II ANOVAs using the dependent variables in Table 1 and the independent variables in Table 2 generally failed to show differences between social and individual training (Supplementary Information). We did observe some non-specific changes in swimming behavior over successive tests, consistent with decreased arousal as the fish become acquainted with the testing tank, such as a decrease in wall following and turn rate.

### 3.2. Training Data

We analyzed data from training trials similarly to data from test trials, with the difference that the test dependent variable is replaced by the trial variable in type II ANOVAs. Additionally, we analyzed the time subjects took to trigger the opening of the door (*T*). ANOVA of triggering time shows no significant effect of social vs. individual condition (Table 5). Thus, zebrafish did not learn to open the door faster, whether learning alone or with the replica. Fish, however, did spend more time close to the correct door as training progressed (Table 6), and showed increased precision in heading toward the correct door (Table 7). Both the preference for the correct door and the precision in heading were stronger when the correct door was on the preferred side of the tank.

**Table 5:** Type II ANOVA table for door triggering time (*T*) during training trials, as a function of training condition, trial, and correct door location.

	**χ**^**2**^	**Df**	**p**
Condition	1.65	1	0.199
Trial	0.68	1	0.411
CorrectDoorLocation	8.88	1	**0.003**
Condition:Trial	0.06	1	0.799
Condition:CorrectDoorLocation	0.09	1	0.762
Trial:CorrectDoorLocation	2.82	1	0.093
Condition:Trial:CorrectDoorLocation	0.22	1	0.638

*Bold values indicate statistically significant results*.

**Table 6:** Type II ANOVA table for the for the preference index (PI) during training trials, as a function of training condition, trial, and correct door location.

	**Chisq**	**Df**	**p**
Condition	3.22	1	0.073
Trial	6.09	1	**0.014**
CorrectDoorLocation	33.09	1	<**0.001**
Condition:Trial	0.68	1	0.408
Condition:CorrectDoorLocation	6.45	1	**0.011**
Trial:CorrectDoorLocation	0.46	1	0.496
Condition:Trial:CorrectDoorLocation	0.02	1	0.885

*Bold values indicate statistically significant results*.

**Table 7:** Type II ANOVA table of heading direction toward the correct door (θ_*C*_) during training trials, as a function of training condition, trial, and correct door location.

	**Chisq**	**Df**	**p**
Condition	0.13	1	0.723
Trial	9.52	1	**0.002**
CorrectDoorLocation	59.87	1	<**0.001**
Condition:Trial	3.19	1	0.074
Condition:CorrectDoorLocation	14.30	1	<**0.001**
Trial:CorrectDoorLocation	1.18	1	0.278
Condition:Trial:CorrectDoorLocation	1.16	1	0.281

*Bold values indicate statistically significant results*.

**Figure 6:**
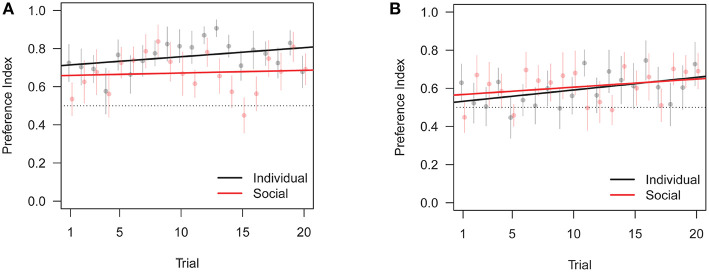
Change in preference index across training trials (PI in Table 1). Bars are standard errors of the mean. **(A)** Preferred correct door location. **(B)** Non-preferred correct door location.

**Figure 7:**
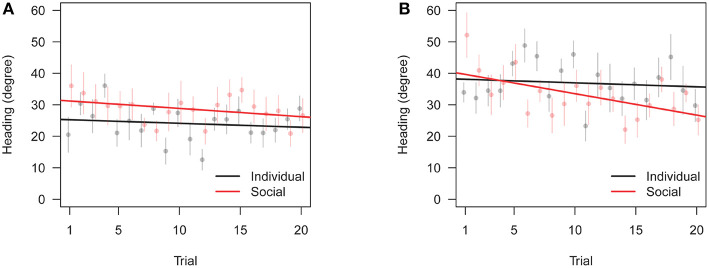
Change in heading precision toward the correct door across training trials (θ_*C*_ in Table 1). Bars are standard errors of the mean. **(A)** Preferred correct door location. **(B)** Non-preferred correct door location.

Similarly to test data, we also found an interaction between training condition and correct door location, in that subjects trained with the replica did slightly worse than subjects trained individually when the correct door was on the preferred side, see Figures 6, 7. Overall, these results are consistent with the focal fish being attracted to locations where it saw the robotic replica, regardless of whether the replica successfully swam through a door.

With respect to potentially aversive effect of the door opening mechanism, we found that focal fish moved away in 80.6 % of the trials when the door started opening (58 out of 72 trials, individual and social learning combined). As a result, we cannot exclude that the door opening might induce a short-term fear reaction on the subjects. The modified preference index, assessing the preference of the fish toward the replica, showed an interaction among the condition, trials, and correct door location. Similar to the analysis of the test data, we found that the tendency of the animals to explore the bottom and top third of the tank where the doors resided increased with the training trials.

The other variables in Table 1 did not differ depending on the training condition, but sometimes we found an effect of the location of the correct door or an interaction between the condition and location of the correct door. For example, trajectory entropy was higher in the social training condition, when the correct door was on the preferred side (Condition×Correct door location interaction: χ^2^_(1)_=43.59, *P* < 0.001), indicating more erratic swimming, consistently with the analogous effect noted above for heading direction. Changes in swimming behavior during training were consistent with those observed during test trials, see above.

## 4. Discussion

We established a novel experimental paradigm, which capitalizes on recent advances in robotics and automated video-tracking to afford fine control of experimental conditions in observational learning. The proposed paradigm features a biologically-inspired zebrafish replica that is controlled by a robotic platform along trajectories, which demonstrate to experimental subjects a route that would allow them to gain proximity to a group of conspecifics. The route consisted of transiting through one of two transparent doors, which automatically opened when the animal spent sufficient time in its proximity. The setup can also be used to investigate individual learning and, as we have done here, to compare individual and social learning.

In addition to its technical innovations, the proposed experimental paradigm appears highly motivating to zebrafish. During the trials, subjects spent considerable time near the transparent partition with the doors, and once they went through the door they swam up to the conspecifics and attempted to interact with them through the one-way glass. While motivating, the setup did not elicit undesired stress responses; experimental subjects swam normally and rarely froze during trials (Supplementary Information). Thus, the proposed robotics-based paradigm could constitute a promising avenue for investigating learning in zebrafish, and can be extended to other organisms.

In our experiment, zebrafish did not open the door faster over successive trials, but they learned to preferentially approach the area near the correct door, and they oriented toward this area more over the course of the experiment. However, fish exposed to the robotic demonstrator did not learn more quickly than fish trained individually, despite having 120 experiences in which the replica approached, opened, and swam through the correct door, and an equal number of experiences in which the incorrect door remained closed when the replica approached it. We thus failed to show observational learning of approach to the correct door location. The only effect of the replica on the subject we found was to reduce the experimental bias toward one of the door locations, which is consistent with the focal fish being attracted to the replica. This failure should not be attributed to a ceiling effect as the task proved difficult enough that social training could have produced a substantial improvement in performance, over the baseline provided by individual training.

Overall, our results suggest that zebrafish social learning may depend on following conspecifics, and thus on experiencing first-hand the relevant stimulus-response contingencies. This hypothesis is consistent with existing demonstrations of social learning in zebrafish (Lindeyer and Reader, 2010) and fish in general (Brown and Laland, 2003), where either following or approach responses were possible. More generally, the hypothesis that social learning requires trying out the behavior to be learned, rather than just observing it in others, is of great relevance to current theory of social learning (Heyes, 2012b; Lind et al., 2019). Previous work has shown that robots can be used to influence the response of animal in longitudinal studies with sequential exposure to robotic stimuli. For example, Locusts (*Locusta migratoria*) learned to escape preferentially on a side, following exposure to a robotic Gecko coming from the opposite side (Romano et al., 2019b). In our case, the robot is used to proxy a trained conspecific that acts as a demonstrator in a social learning task, while in the study by Romano et al. (2019b) a robotic predator served as aversive stimulus to condition the subjects spatially. Our experimental paradigm could serve as inspiration to design similar studies in other species.

The work that most closely resembles ours is, perhaps, that of Dugatkin and coworkers on mate choice copying in female guppies (Dugatkin and Godin, 1992, 1993; Godin et al., 2005). In these experiments, a female subject could observe another female approaching one of two males, which resulted in the subject subsequently preferring to approach the same male. This result is seemingly at odds with ours, for which several explanations are possible. First, the capacity for observational learning may be dependent on which behavior system is engaged. Because a single mate choice is likely more important for fitness than a single choice of swimming direction, mate choice decisions may have evolved to take into account social information to a larger extent [indeed, Dugatkin and Godin (1993) showed that it is mainly inexperienced females that copy the preferences of others]. Second, it is possible that the subjects of Dugatkin and coworkers learned a preference for a stimulus (a male) rather than an approach response, which then resulted in approach because of a hardwired predisposition to approach males. In our experiment, however, there was no conspicuous visual stimulus for which a preference could be learned. Lastly, the learning observed by Dugatkin and coworkers may have been driven by responses performed during the observation phase. Female subjects, in fact, had to choose between two males at the opposite ends of a tank. Observing the female demonstrator would thus have biased the subject to turn toward one end of the tank, which may have been instrumental in establishing the preference for swimming in that direction once this became possible. In our experimental setup, on the other hand, the two doors were both in front of the subject, and the scope for orienting differentially toward one or the other was much more limited.

Related work by Webster and Laland involved food, which could offer a more motivating stimulus than a shoal of conspecifics. In these studies, the demonstrator also displayed feeding behavior, which is likely to convey additional information, compared to swimming toward a particular location. Furthermore, with food patches, Webster and Laland (2017) demonstrated the ability of both social and non-social species to use social information in the determination of the better patch. Nine-spined Stickleback (*Pungitius pungitius*) were shown to be more likely to travel toward the location where they had previously observed other individuals feeding (Webster and Laland, 2015), while social learning was more likely observed when predation risk was higher in Minnows (*Phoxinus phoxinus*) (Webster and Laland, 2008). The difference in results between our experiment and the above-mentioned studies suggests many opportunities for further investigation.

Additional caution in drawing conclusions about zebrafish social learning is advised given that our study is the first attempt to disentangle observational learning from following, and given the novelty of our experimental paradigm. For example, we cannot exclude that a live zebrafish would have been a more effective demonstrator, although in previous work we established that zebrafish associate with the replica and with live conspecifics to similar extents, when given the choice (Ruberto et al., 2016, 2017; Kim et al., 2018). The replica also demonstrated the door-opening behavior with much more precision and consistency than a live fish could have done. Our task, however, might have been more difficult than other tasks in the zebrafish learning literature, since it required experimental subjects to approach a small area and station there for 3 s out of any 5 s window. This behavior is more complex than behaviors investigated in other studies, in which subjects simply had to swim in one or another direction without a time requirement (Bilotta et al., 2005; Xu et al., 2007; Pather and Gerlai, 2009; Sison and Gerlai, 2010; Morin et al., 2013). Our task also lacked salient visual cues distinguishing the correct door from the incorrect one, although previous work suggests that zebrafish can improve substantially in a spatial discrimination in fewer than 10 trials (Arthur and Levin, 2001). Finally, our task required the fish to remember which door the replica had swam through, although this memory had to be maintained only for a few seconds. To evaluate whether observational learning could be more effective in different circumstances, we will perform further experiments with visually marked doors, a reduced time to trigger door opening, and a shorter interval between the replica crossing the door and the subject being released. We will also evaluate whether allowing zebrafish to follow the robotic replica leads to better learning.

While this is the first robotics-based setup for zebrafish learning, a few previous efforts have explored other automation techniques. For example, Pather and Gerlai (2009) utilized computer-animated images of zebrafish as rewarding stimuli in a shuttle box task, while (Hicks et al., 2006) used real-time video tracking to deliver rewarding or punishing stimuli, in the form of a change in illumination and brief electric shock, Gerlai et al. (2009) showed that zebrafish react to computerized images of a predator, and Fangmeier et al. (2018) demonstrated the possibility to use automated video stimulus to quantify behavioral traits in zebrafish. Here, we took a significant step forward by combining engineered stimuli with real-time control, affording the possibility of maneuvering them in the entire experimental tank. For example, compared to the experimental modifications in Hicks et al. (2006), our approach offers an additional independent variable to explore social learning, by enabling, for the first time, high-precision demonstration through a biologically-inspired replica.

The potentially negative effects on learning of the door opening mechanism and the presence of the robot seem to be limited. Although focal fish might have initially displayed an aversive response toward the door as it started opening, they eventually went through the door to interact with the fish shoal. The short-term avoidance reaction is likely due to the mechanical noise from the door movement and the concurrent water motion, which did not last long enough to significantly affect their motivation to join the shoal. The modified preference index significantly decreased in both training and test trials, indicating that, over time, the focal fish increasingly preferred to spend time in the vicinity of the doors rather than close to the replica that was visible through the partition. Thus, the potential attraction toward the replica did not significantly reduce fish motivation to explore the doors.

In conclusion, we have presented a novel robotics-based experimental paradigm that enables us to study both social and individual learning in zebrafish, with many possible variations in experimental parameters. Beyond zebrafish, the setup can be adapted to investigate social learning in other animal species for which ethorobotics-based approaches have been previously explored, including, other fish species, such as guppies (Landgraf et al., 2016; Bierbach et al., 2018a) and mollies (Bierbach et al., 2018b), insects, such as bees (Landgraf et al., 2018) and cockroaches (Halloy et al., 2007), and mammals, such as tree squirrels (Partan et al., 2009), dogs (Kubinyi et al., 2004), and rats (Takanishi et al., 1998), and even to invertebrates such as cephalopods, for example by using prey items as motivating stimulus rather than a shoal of conspecifics. The data presented above suggest that our paradigm has the potential to contribute new knowledge to the experimental analysis of learning in fish and other aquatic animals.

## Data Availability

The supporting data and codes to reproduce the analysis and results of the paper has been uploaded as Supplementary Information.

## Ethics Statement

All animal procedures were approved by the University Animal Welfare Committee of New York University under protocol number 13–1424.

## Author Contributions

SG and MP designed the research. YY designed and developed the experimental setup. YY and RC conducted the experiments. YY wrote a first draft of the manuscript and RC offered comments. SG and MP wrote the final draft. All the authors analyzed the data, discussed the results, and reviewed and approved the final draft.

### Conflict of Interest Statement

The authors declare that the research was conducted in the absence of any commercial or financial relationships that could be construed as a potential conflict of interest.

## Supplementary Material

Video S1 Exemplary video of individual training.

Video S2 Exemplary video of social training.
