# Supplementary material for: A Comparison of Individual Learning and Social Learning in Zebrafish Through an Ethorobotics Approach
Source: Front Robot AI. 2019 Aug 14;6:71. doi: 10.3389/frobt.2019.00071 (PMC7805697; doi:10.3389/frobt.2019.00071)
Supplement: Supplementary file 1 [file Data_Sheet_1.pdf]

Supplementary Material

1 SUPPLEMENTARY DATA

2 INTRODUCTION

This document replicates the analyses in the companion paper, and includes further analysis of zebrafish behavior. Section 4 provides an orientation to the data fields in files TestData.csv and TrainingData.csv, which contain all data analyzed in the companion paper and below. Sections 5 to 11 reproduce the analysis in the companion paper, with some additional details. Later sections show ANOVA tables for other behavioral measures, according to the independent variables of condition (social or individual training), correct door location (preferred or non-preferred), and test/trial number.

All ANOVAs refer to linear mixed models with subject as a random factor; p values have been computed with the `Anova` function of the `car` package within the R statistical environment (see main paper for references).

3 CONTENTS OF DATA PACKAGE

TestData.csv: Data collected during test trials.
TrainingData.csv: Data collected during training trials.
data sheet 1.pdf: This file.
YangEtAl-ZebrafishLearning.org: Emacs Org-mode source code for the analysis and for this file (Schulte et al., 2012; Dominik, 2010)
YangEtAl-ZebrafishLearning.R:R code for data analysis, extracted from YangEtAl-ZebrafishLearning.org.

4 DATA DESCRIPTION

The columns in `TestData.csv` and `TrainingData.csv` describe test and training conditions and results. Column meaning is described in the following table.

Table S1: Description of data columns.

	Column name	Description
1	FishId	Subject Id within a batch
2	Sex	Subject sex
3	CorrectSide	Side of correct door from the point of view of the fish shoal
4	Trial/Test	Trial or test number
5	Time	Time and date of experiment
6	TrigTime	Time at which door opening was triggered
7	PI	Preference Index

Continued on next page

Continued from previous page

	Column name	Description
8	TimeR	Time spent in a 6×6cm region in front of the right door (from the point of view of an observer facing the subject)
9	TimeL	Time spent in proximity of the left door
10	TimeRest	Time spent elsewhere, until the subject triggers the door or the trial ends
11	Condition	Experimental condition (social or individual training)
12	Batch	Experimental batch (1 or 2)
13	GlobalId	Subject Id unique to the whole data set
14	Entropy	Entropy of the trajectory
15	AveSpeed	Average speed
16	AvePeakSpeed	Average peak speed across all test or training trials
17	AveTravel	Distance traveled
18	AveCorrDoorDistance	Average distance to the correct door
19	AveWronDoorDistance	Average distance to the wrong door
20	AveDoorDisRatio	Ratio of distances to the correct and wrong door
21	AveRobotDistance	Average distance from the final resting point of the robotic replica
22	WallTime1	Time spent in proximity of wall 1
23	WallTime2	Time spent in proximity of wall 2
24	WallTime3	Time spent in proximity of wall 3
25	WallTime4	Time spent in proximity of wall 4
26	WallTimeAll	Time spent in proximity of any wall
27	FreezingTime	Time spent moving more slowly than 2 cm/s
28	MovingTime	Time spent moving faster than 2 cm/s
29	AveHeading	Average subject's heading angle (0 represents heading toward the section housing conspecifics)
30	AveHeadingDoorAngle	Average angle between subject heading and the direction from the subject's centroid to the correct door
31	AveAngularSpeed	Average turn rate
32	AvePeakAngSpeed	Average peak turn rate
33	AveAcceleration	Average acceleration across test or training trials
34	AvePeakAcceler	Average peak acceleration across test or training trials
35	CorrectDoorLocation	Location of the correct door in the frame of reference of the experimental room
36	Heading	Absolute value of AveHeadingDoorAngle, transformed from radians to degrees
37	Avoidance	Avoidance response for the door after it opened
38	PI_m	Modified preference index for the replica

5 PREFERENCE INDEX AT TEST

ANOVA of Preference index shows improvement across tests, but independent of social or individual training (Fig. S1), and primarily limited to the preferred door location (Fig. S2).

Table S2. ANOVA of preference index. Here and in what follows bold number numbers identify statistically significant results.

	Chisq	Df	Pr(>Chisq)
Condition	0.82	1	0.364
CorrectDoorLocation	1.44	1	0.230
Test	10.44	2	**0.005**
Condition:CorrectDoorLocation	0.91	1	0.339
Condition:Test	2.10	2	0.350
CorrectDoorLocation:Test	12.89	2	**0.002**
Condition:CorrectDoorLocation:Test	1.56	2	0.459

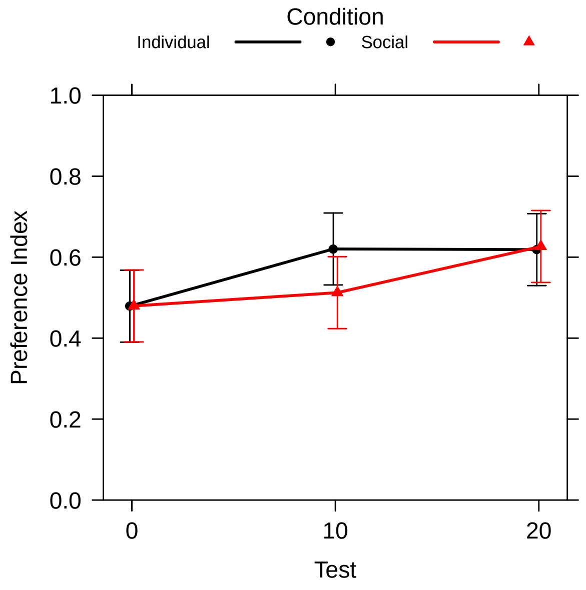

Figure S1: Preference index at test: non-significant difference of social vs. individual training. Bars are 95% confidence intervals.

5.1 Variability

Levene's test shows no significant difference in variability of preference index in the subject groups defined by different levels of independent variables in the ANOVA in Table S2:

```
Levene's test for homogeneity of variance (center = median)
      Df F value Pr(>F)
group 11  1.1343 0.3437
      96
```

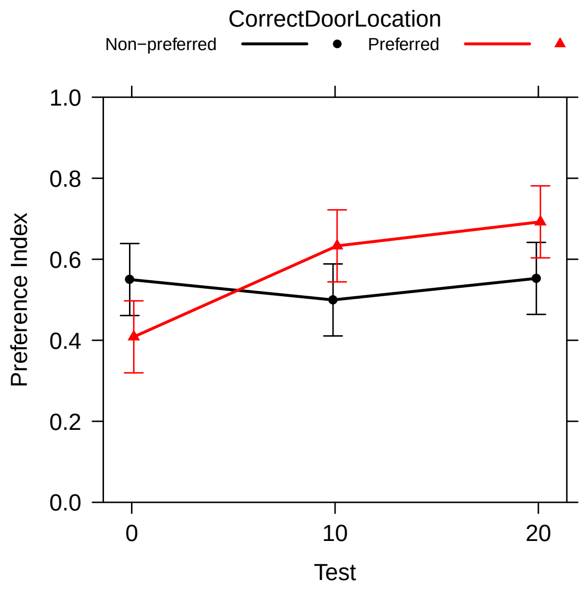

Figure S2: Preference index: significant interaction between correct door location and test. Bars are 95% confidence intervals.

6 HEADING AT TEST

ANOVA of heading direction at test shows the same pattern as Preference Index (Table S3, Fig. S3, Fig. S4).

Table S3. ANOVA of heading direction.

	Chisq	Df	Pr(>Chisq)
Condition	1.55	1	0.213
CorrectDoorLocation	0.39	1	0.535
Test	14.30	2	**0.001**
Condition:CorrectDoorLocation	8.02	1	**0.005**
Condition:Test	0.05	2	0.975
CorrectDoorLocation:Test	8.45	2	**0.015**
Condition:CorrectDoorLocation:Test	0.07	2	0.964

6.1 Variability

Levene's test shows no difference in variability in heading direction in the ANOVA in Table S3:

```
Levene's test for homogeneity of variance (center = median)
      Df F value Pr(>F)
group 11  0.3167 0.9806
      96
```

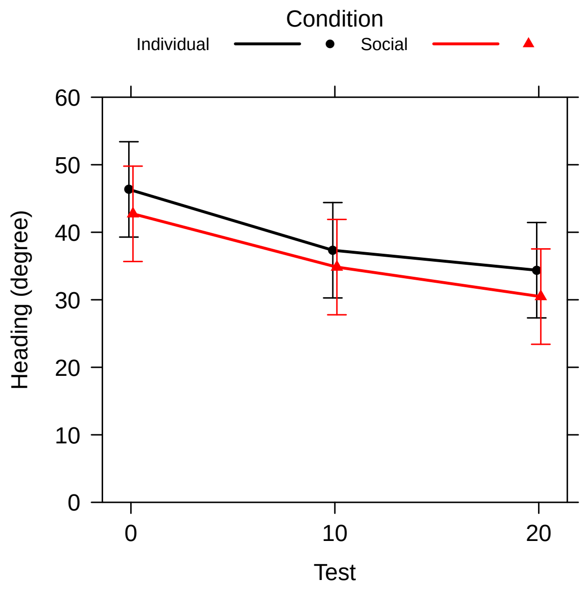

Figure S3: Heading direction at test: showing non-significant difference between social vs. individual training. Bars are 95% confidence intervals.

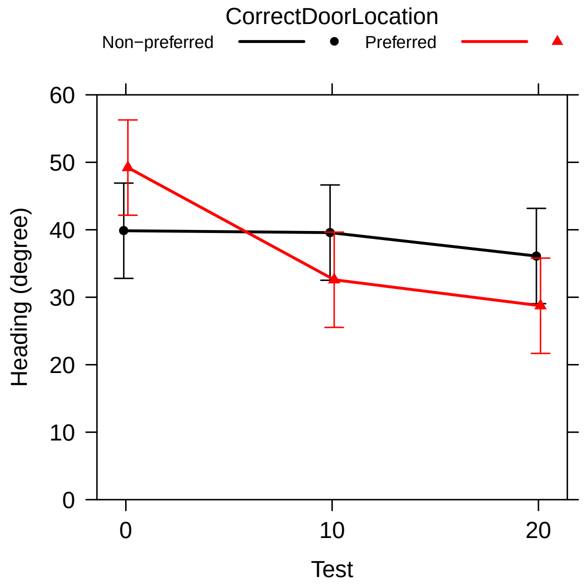

Figure S4: Heading direction at test: significant interaction between test and location of the correct door. Bars are 95% confidence intervals.

7 DOOR TRIGGERING TIME DURING TRAINING

ANOVA of door triggering time during training shows a significant effect of correct door location but no effect of training condition (social or individual) or trial number (Fig. S5).

Table S4. ANOVA of door triggering time.

	Chisq	Df	Pr(>Chisq)
Condition	1.65	1	0.199
Trial	0.68	1	0.411
CorrectDoorLocation	8.88	1	**0.003**
Condition:Trial	0.06	1	0.799
Condition:CorrectDoorLocation	0.09	1	0.762
Trial:CorrectDoorLocation	2.82	1	0.093
Condition:Trial:CorrectDoorLocation	0.22	1	0.638

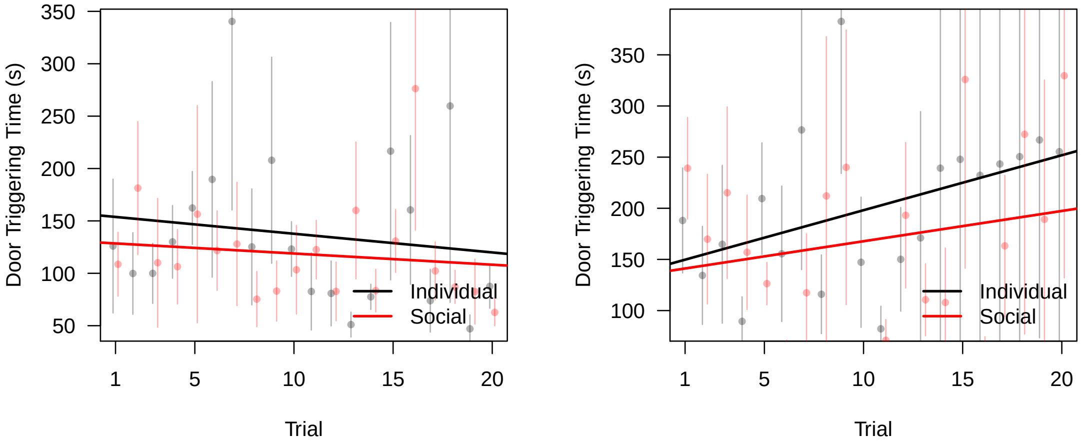

Figure S5: Changes in door triggering time with training, when the correct door is in the preferred location (left panel) or the non-preferred location (right-panel). Bars are standard errors.

7.1 Variability

Levene's test suggests a difference in variability of door triggering time in the subject groups defined by the condition and door location independent variables in the ANOVA in Table S4:

```
Levene's test for homogeneity of variance (center = median)
       Df F value  Pr(>F)
group   3  3.5393 0.01444 *
      716
---
Signif. codes:  0 '***' 0.001 '**' 0.01 '*' 0.05 '.' 0.1 ' ' 1
```

The difference lies primarily in higher variability when the door is in the preferred versus the non-preferred location (Fig. S6):

```
    Condition CorrectDoorLocation var(TrigTime)
```

```
1: Individual       Non-preferred     145305.97
2: Individual           Preferred      54733.68
3:     Social       Non-preferred      90466.95
4:     Social           Preferred      24932.90
```

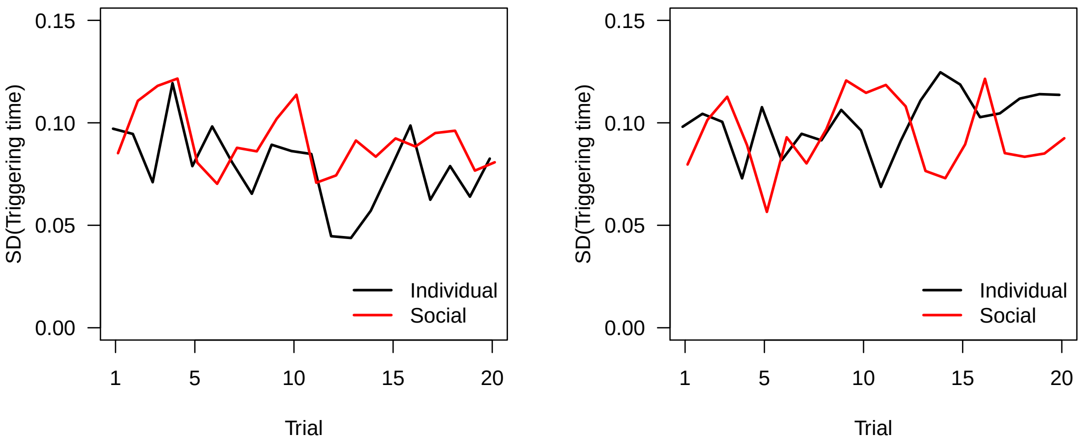

Figure S6: Standard deviation of triggering time through training, when the correct door is in the preferred location (left panel) or the non-preferred location (right-panel).

8 PREFERENCE INDEX DURING TRAINING

ANOVA of preference index shows an effect of location of the correct door, an effect of trials due to increasing preference, and an interaction between door location and condition.

Table S5. ANOVA of preference index during training.

	Chisq	Df	Pr(>Chisq)
Condition	3.22	1	0.073
Trial	6.09	1	**0.014**
CorrectDoorLocation	33.09	1	$<$**0.001**
Condition:Trial	0.68	1	0.408
Condition:CorrectDoorLocation	6.45	1	**0.011**
Trial:CorrectDoorLocation	0.46	1	0.496
Condition:Trial:CorrectDoorLocation	0.02	1	0.885

Visualization of the data shows that social training results in a smaller preference than individual training, although the difference in not significant (Fig. S7).

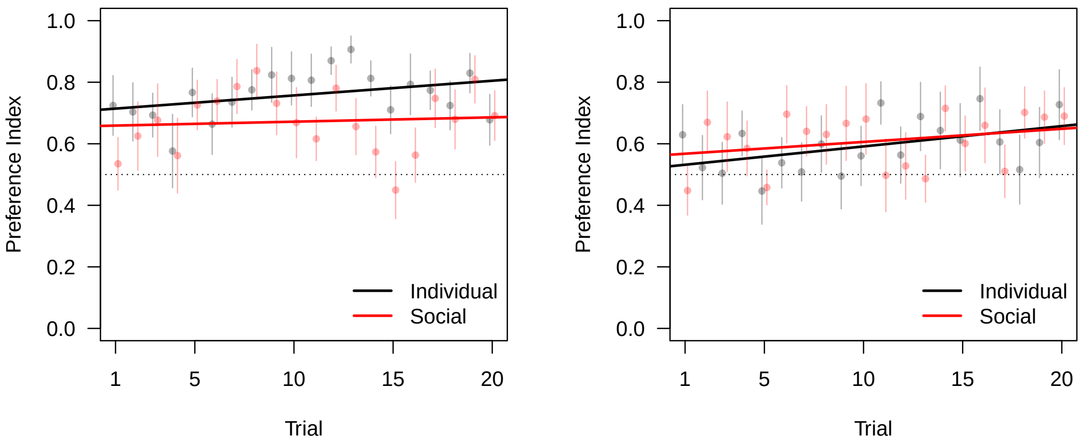

Figure S7: Changes in preference index with training, when the correct door is in the preferred location (left panel) or the non-preferred location (right-panel).

9 HEADING DURING TRAINING

ANOVA of heading during training shows a significant improvement over trials, a significant effect of correct door location, and an interaction between condition and correct door location (Table S6, Fig. S8). The latter signifies that social training was better than individual training when the door was on the preferred side, and worse when the door was on the non-preferred side.

Table S6. ANOVA of Heading during training.

	Chisq	Df	Pr(>Chisq)
Condition	0.13	1	0.723
Trial	9.52	1	**0.002**
CorrectDoorLocation	59.87	1	<**0.001**
Condition:Trial	3.19	1	0.074
Condition:CorrectDoorLocation	14.30	1	<**0.001**
Trial:CorrectDoorLocation	1.18	1	0.278
Condition:Trial:CorrectDoorLocation	1.16	1	0.281

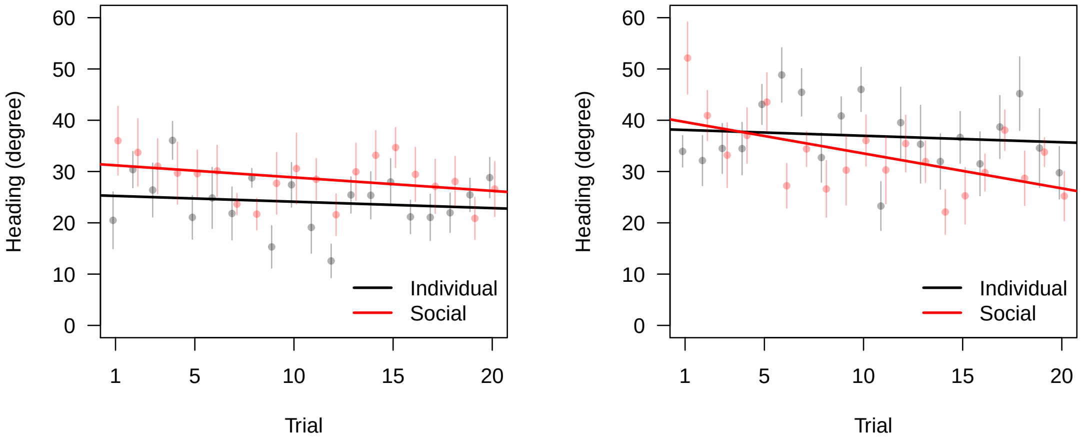

Figure S8: Changes in Heading with training, when the correct door is in the preferred location (left panel) or the non-preferred location (right-panel).

10 MODIFIED PREFERENCE INDEX FOR THE REPLICA AT TEST

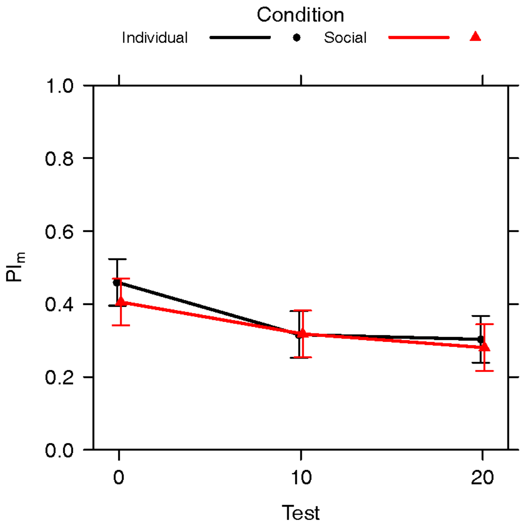

Figure S9: Modified preference index at test: significant on test. Bars are 95 % confidence intervals.

Table S7. ANOVA of PI_m at test trials.

	Chisq	Df	Pr(>Chisq)
Condition	1.06	1	0.303
Test	25.45	2	<**0.001**
CorrectDoorLocation	0.00	1	0.982
Condition:Test	0.90	2	0.637
Condition:CorrectDoorLocation	3.39	1	0.066
Test:CorrectDoorLocation	0.28	2	0.870
Condition:Test:CorrectDoorLocation	0.41	2	0.815

11 MODIFIED PREFERENCE INDEX FOR THE REPLICA DURING TRAINING

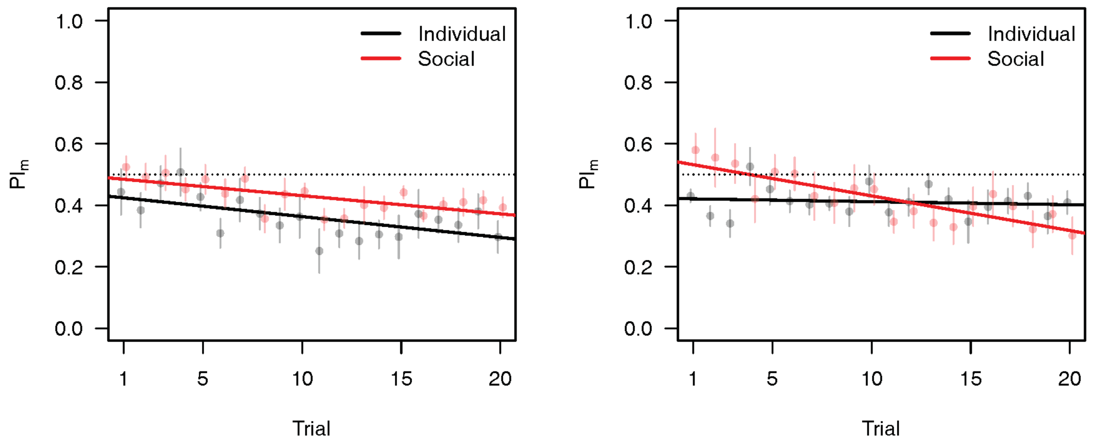

Figure S10: Modified preference index during training, when the correct door is in the preferred location (left panel) or the non-preferred location (right-panel).

Table S8. ANOVA of PI_m during training.

	Chisq	Df	Pr(>Chisq)
Condition	11.95	1	**0.001**
Trial	39.74	1	<**0.001**
CorrectDoorLocation	4.17	1	0.041
Condition:Trial	5.64	1	**0.018**
Condition:CorrectDoorLocation	5.76	1	**0.016**
Trial:CorrectDoorLocation	0.01	1	0.916
Condition:Trial:CorrectDoorLocation	7.88	1	**0.005**

12 OTHER VARIABLES AT TEST

The following ANOVA tables relate the independent variables of condition (social/individual), correct door location (preferred/non-preferred), and test to the dependent variables listed in Section 4 that were not discussed above. The results are typically non-significant, especially for the difference between social and individual training.

The code for the following ANOVA is generated automatically (see R enclosed script), and non-significant interactions are not removed.

Table S9. ANOVA of Entropy across test trials.

	Chisq	Df	Pr(>Chisq)
Condition	0.63	1	0.429
Test	3.24	2	0.198
CorrectDoorLocation	4.80	1	0.029
Condition:Test	1.37	2	0.504
Condition:CorrectDoorLocation	0.62	1	0.432
Test:CorrectDoorLocation	1.73	2	0.421
Condition:Test:CorrectDoorLocation	0.60	2	0.740

Table S10. ANOVA of AveSpeed across test trials.

	Chisq	Df	Pr(>Chisq)
Condition	0.62	1	0.432
Test	2.31	2	0.315
CorrectDoorLocation	0.16	1	0.694
Condition:Test	1.04	2	0.594
Condition:CorrectDoorLocation	0.06	1	0.807
Test:CorrectDoorLocation	1.06	2	0.589
Condition:Test:CorrectDoorLocation	0.14	2	0.934

Table S11. ANOVA of AvePeakSpeed across test trials.

	Chisq	Df	Pr(>Chisq)
Condition	0.01	1	0.916
Test	0.17	2	0.917
CorrectDoorLocation	0.59	1	0.444
Condition:Test	2.15	2	0.341
Condition:CorrectDoorLocation	1.31	1	0.252
Test:CorrectDoorLocation	1.35	2	0.508
Condition:Test:CorrectDoorLocation	1.02	2	0.602

Table S12. ANOVA of AveTravel across test trials.

	Chisq	Df	Pr(>Chisq)
Condition	0.62	1	0.430
Test	2.30	2	0.317
CorrectDoorLocation	0.15	1	0.696
Condition:Test	1.04	2	0.593
Condition:CorrectDoorLocation	0.06	1	0.809
Test:CorrectDoorLocation	1.05	2	0.591
Condition:Test:CorrectDoorLocation	0.14	2	0.932

Table S13. ANOVA of AveCorrDoorDistance across test trials.

	Chisq	Df	Pr(>Chisq)
Condition	0.48	1	0.488
Test	1.09	2	0.579
CorrectDoorLocation	0.00	1	0.989
Condition:Test	0.45	2	0.800
Condition:CorrectDoorLocation	0.12	1	0.734
Test:CorrectDoorLocation	1.20	2	0.550
Condition:Test:CorrectDoorLocation	1.84	2	0.399

Table S14. ANOVA of AveWronDoorDistance across test trials.

	Chisq	Df	Pr(>Chisq)
Condition	1.21	1	0.271
Test	16.12	2	0.000
CorrectDoorLocation	4.40	1	0.036
Condition:Test	1.79	2	0.408
Condition:CorrectDoorLocation	3.06	1	0.080
Test:CorrectDoorLocation	10.94	2	0.004
Condition:Test:CorrectDoorLocation	2.58	2	0.276

Table S15. ANOVA of AveDoorDisRatio across test trials.

	Chisq	Df	Pr(>Chisq)
Condition	0.01	1	0.933
Test	7.34	2	0.026
CorrectDoorLocation	1.89	1	0.169
Condition:Test	0.52	2	0.771
Condition:CorrectDoorLocation	1.96	1	0.162
Test:CorrectDoorLocation	16.56	2	0.000
Condition:Test:CorrectDoorLocation	0.50	2	0.779

Table S16. ANOVA of AveRobotDistance across test trials.

	Chisq	Df	Pr(>Chisq)
Condition	1.28	1	0.258
Test	12.55	2	0.002
CorrectDoorLocation	1.99	1	0.159
Condition:Test	1.08	2	0.582
Condition:CorrectDoorLocation	0.65	1	0.422
Test:CorrectDoorLocation	2.30	2	0.317
Condition:Test:CorrectDoorLocation	3.05	2	0.218

Table S17. ANOVA of WallTime1 across test trials.

	Chisq	Df	Pr(>Chisq)
Condition	2.22	1	0.136
Test	22.56	2	0.000
CorrectDoorLocation	2.86	1	0.091
Condition:Test	1.27	2	0.531
Condition:CorrectDoorLocation	0.72	1	0.395
Test:CorrectDoorLocation	0.96	2	0.618
Condition:Test:CorrectDoorLocation	1.47	2	0.479

Table S18. ANOVA of WallTime2 across test trials.

	Chisq	Df	Pr(>Chisq)
Condition	9.79	1	0.002
Test	2.59	2	0.274
CorrectDoorLocation	0.18	1	0.667
Condition:Test	2.00	2	0.367
Condition:CorrectDoorLocation	0.06	1	0.814
Test:CorrectDoorLocation	1.46	2	0.482
Condition:Test:CorrectDoorLocation	0.93	2	0.627

Table S19. ANOVA of WallTime3 across test trials.

	Chisq	Df	Pr(>Chisq)
Condition	2.56	1	0.109
Test	2.39	2	0.303
CorrectDoorLocation	2.24	1	0.134
Condition:Test	1.83	2	0.400
Condition:CorrectDoorLocation	1.18	1	0.277
Test:CorrectDoorLocation	2.30	2	0.317
Condition:Test:CorrectDoorLocation	3.41	2	0.182

Table S20. ANOVA of WallTime4 across test trials.

	Chisq	Df	Pr(>Chisq)
Condition	1.98	1	0.160
Test	0.83	2	0.659
CorrectDoorLocation	1.10	1	0.294
Condition:Test	5.19	2	0.075
Condition:CorrectDoorLocation	0.25	1	0.614
Test:CorrectDoorLocation	0.75	2	0.687
Condition:Test:CorrectDoorLocation	1.26	2	0.532

Table S21. ANOVA of WallTimeAll across test trials.

	Chisq	Df	Pr(>Chisq)
Condition	0.12	1	0.731
Test	16.22	2	0.000
CorrectDoorLocation	3.71	1	0.054
Condition:Test	3.21	2	0.201
Condition:CorrectDoorLocation	0.12	1	0.725
Test:CorrectDoorLocation	0.45	2	0.799
Condition:Test:CorrectDoorLocation	2.11	2	0.348

Table S22. ANOVA of FreezingTime across test trials.

	Chisq	Df	Pr(>Chisq)
Condition	0.00	1	0.957
Test	3.43	2	0.180
CorrectDoorLocation	0.50	1	0.479
Condition:Test	0.68	2	0.711
Condition:CorrectDoorLocation	3.58	1	0.059
Test:CorrectDoorLocation	0.83	2	0.661
Condition:Test:CorrectDoorLocation	1.89	2	0.389

Table S23. ANOVA of MovingTime across test trials.

	Chisq	Df	Pr(>Chisq)
Condition	0.00	1	0.961
Test	3.41	2	0.182
CorrectDoorLocation	0.51	1	0.477
Condition:Test	0.69	2	0.710
Condition:CorrectDoorLocation	3.56	1	0.059
Test:CorrectDoorLocation	0.82	2	0.664
Condition:Test:CorrectDoorLocation	1.90	2	0.386

Table S24. ANOVA of AveHeading across test trials.

	Chisq	Df	Pr(>Chisq)
Condition	6.88	1	0.009
Test	5.97	2	0.051
CorrectDoorLocation	5.16	1	0.023
Condition:Test	0.52	2	0.772
Condition:CorrectDoorLocation	2.36	1	0.125
Test:CorrectDoorLocation	5.93	2	0.052
Condition:Test:CorrectDoorLocation	0.69	2	0.709

Table S25. ANOVA of AveHeadingDoorAngle across test trials.

	Chisq	Df	Pr(>Chisq)
Condition	5.37	1	0.021
Test	0.63	2	0.729
CorrectDoorLocation	0.19	1	0.659
Condition:Test	0.84	2	0.658
Condition:CorrectDoorLocation	0.24	1	0.626
Test:CorrectDoorLocation	0.82	2	0.664
Condition:Test:CorrectDoorLocation	1.04	2	0.596

Table S26. ANOVA of AveAngularSpeed across test trials.

	Chisq	Df	Pr(>Chisq)
Condition	1.08	1	0.299
Test	28.85	2	0.000
CorrectDoorLocation	2.80	1	0.094
Condition:Test	2.18	2	0.337
Condition:CorrectDoorLocation	1.96	1	0.162
Test:CorrectDoorLocation	0.22	2	0.895
Condition:Test:CorrectDoorLocation	1.12	2	0.570

Table S27. ANOVA of AvePeakAngSpeed across test trials.

	Chisq	Df	Pr(>Chisq)
Condition	1.06	1	0.302
Test	32.66	2	0.000
CorrectDoorLocation	3.41	1	0.065
Condition:Test	2.18	2	0.336
Condition:CorrectDoorLocation	1.94	1	0.164
Test:CorrectDoorLocation	0.05	2	0.975
Condition:Test:CorrectDoorLocation	0.97	2	0.616

Table S28. ANOVA of AveAcceleration across test trials.

	Chisq	Df	Pr(>Chisq)
Condition	0.17	1	0.678
Test	12.84	2	0.002
CorrectDoorLocation	0.92	1	0.338
Condition:Test	1.47	2	0.478
Condition:CorrectDoorLocation	0.68	1	0.409
Test:CorrectDoorLocation	0.59	2	0.744
Condition:Test:CorrectDoorLocation	0.30	2	0.862

Table S29. ANOVA of AvePeakAcceler across test trials.

	Chisq	Df	Pr(>Chisq)
Condition	0.50	1	0.479
Test	6.38	2	0.041
CorrectDoorLocation	1.08	1	0.298
Condition:Test	4.24	2	0.120
Condition:CorrectDoorLocation	0.00	1	0.949
Test:CorrectDoorLocation	0.78	2	0.678
Condition:Test:CorrectDoorLocation	0.60	2	0.741

Table S30. ANOVA of Heading across test trials.

	Chisq	Df	Pr(>Chisq)
Condition	1.55	1	0.213
Test	14.30	2	0.001
CorrectDoorLocation	0.39	1	0.535
Condition:Test	0.05	2	0.975
Condition:CorrectDoorLocation	8.02	1	0.005
Test:CorrectDoorLocation	8.45	2	0.015
Condition:Test:CorrectDoorLocation	0.07	2	0.964

13 OTHER VARIABLES DURING TRAINING

The following ANOVA tables relate the independent variables of condition (social/individual), correct door location (preferred/non-preferred), and trial to the dependent variables listed in Section 4 that were not discussed above. The results are typically non-significant, especially for the difference between social and individual training.

The code for the following ANOVA is generated automatically (see R enclosed script), and non-significant interactions are not removed.

Table S31. ANOVA of Entropy during training

	Chisq	Df	Pr(>Chisq)
Condition	1.58	1	0.209
Trial	0.25	1	0.617
CorrectDoorLocation	31.96	1	0.000
Condition:Trial	2.34	1	0.126
Condition:CorrectDoorLocation	43.59	1	0.000
Trial:CorrectDoorLocation	7.66	1	0.006
Condition:Trial:CorrectDoorLocation	0.34	1	0.557

Table S32. ANOVA of AveSpeed during training

	Chisq	Df	Pr(>Chisq)
Condition	1.41	1	0.235
Trial	10.67	1	0.001
CorrectDoorLocation	0.05	1	0.828
Condition:Trial	0.63	1	0.426
Condition:CorrectDoorLocation	0.34	1	0.559
Trial:CorrectDoorLocation	2.53	1	0.112
Condition:Trial:CorrectDoorLocation	2.11	1	0.146

Table S33. ANOVA of AvePeakSpeed during training

	Chisq	Df	Pr(>Chisq)
Condition	0.00	1	0.947
Trial	7.03	1	0.008
CorrectDoorLocation	9.19	1	0.002
Condition:Trial	0.04	1	0.839
Condition:CorrectDoorLocation	13.32	1	0.000
Trial:CorrectDoorLocation	0.09	1	0.766
Condition:Trial:CorrectDoorLocation	0.66	1	0.417

Table S34. ANOVA of AveTravel during training

	Chisq	Df	Pr(>Chisq)
Condition	2.84	1	0.092
Trial	4.21	1	0.040
CorrectDoorLocation	1.50	1	0.221
Condition:Trial	2.54	1	0.111
Condition:CorrectDoorLocation	3.04	1	0.081
Trial:CorrectDoorLocation	0.82	1	0.364
Condition:Trial:CorrectDoorLocation	0.03	1	0.857

Table S35. ANOVA of AveCorrDoorDistance during training

	Chisq	Df	Pr(>Chisq)
Condition	7.46	1	0.006
Trial	2.08	1	0.150
CorrectDoorLocation	2.21	1	0.137
Condition:Trial	4.45	1	0.035
Condition:CorrectDoorLocation	5.20	1	0.023
Trial:CorrectDoorLocation	1.14	1	0.286
Condition:Trial:CorrectDoorLocation	1.40	1	0.237

Table S36. ANOVA of AveWronDoorDistance during training

	Chisq	Df	Pr(>Chisq)
Condition	16.48	1	0.000
Trial	2.35	1	0.125
CorrectDoorLocation	118.62	1	0.000
Condition:Trial	3.58	1	0.058
Condition:CorrectDoorLocation	3.93	1	0.047
Trial:CorrectDoorLocation	0.02	1	0.884
Condition:Trial:CorrectDoorLocation	0.67	1	0.414

Table S37. ANOVA of AveDoorDisRatio during training

	Chisq	Df	Pr(>Chisq)
Condition	0.33	1	0.568
Trial	0.94	1	0.331
CorrectDoorLocation	6.51	1	0.011
Condition:Trial	0.49	1	0.486
Condition:CorrectDoorLocation	2.23	1	0.135
Trial:CorrectDoorLocation	0.61	1	0.435
Condition:Trial:CorrectDoorLocation	0.22	1	0.637

Table S38. ANOVA of AveRobotDistance during training

	Chisq	Df	Pr(>Chisq)
Condition	19.29	1	0.000
Trial	0.01	1	0.928
CorrectDoorLocation	60.70	1	0.000
Condition:Trial	7.38	1	0.007
Condition:CorrectDoorLocation	0.05	1	0.823
Trial:CorrectDoorLocation	0.69	1	0.405
Condition:Trial:CorrectDoorLocation	0.09	1	0.764

Table S39. ANOVA of WallTime1 during training

	Chisq	Df	Pr(>Chisq)
Condition	1.66	1	0.198
Trial	11.91	1	0.001
CorrectDoorLocation	3.02	1	0.082
Condition:Trial	1.16	1	0.282
Condition:CorrectDoorLocation	2.59	1	0.108
Trial:CorrectDoorLocation	0.77	1	0.382
Condition:Trial:CorrectDoorLocation	0.09	1	0.764

Table S40. ANOVA of WallTime2 during training

	Chisq	Df	Pr(>Chisq)
Condition	6.34	1	0.012
Trial	3.79	1	0.052
CorrectDoorLocation	4.00	1	0.046
Condition:Trial	4.52	1	0.033
Condition:CorrectDoorLocation	3.75	1	0.053
Trial:CorrectDoorLocation	3.20	1	0.074
Condition:Trial:CorrectDoorLocation	3.06	1	0.080

Table S41. ANOVA of WallTime3 during training

	Chisq	Df	Pr(>Chisq)
Condition	0.11	1	0.743
Trial	0.04	1	0.845
CorrectDoorLocation	0.08	1	0.783
Condition:Trial	0.78	1	0.376
Condition:CorrectDoorLocation	0.02	1	0.890
Trial:CorrectDoorLocation	0.04	1	0.842
Condition:Trial:CorrectDoorLocation	0.50	1	0.481

Table S42. ANOVA of WallTime4 during training

	Chisq	Df	Pr(>Chisq)
Condition	9.45	1	0.002
Trial	0.10	1	0.750
CorrectDoorLocation	6.06	1	0.014
Condition:Trial	0.02	1	0.880
Condition:CorrectDoorLocation	2.82	1	0.093
Trial:CorrectDoorLocation	0.00	1	0.947
Condition:Trial:CorrectDoorLocation	0.03	1	0.872

Table S43. ANOVA of WallTimeAll during training

	Chisq	Df	Pr(>Chisq)
Condition	2.27	1	0.131
Trial	0.00	1	0.996
CorrectDoorLocation	1.98	1	0.159
Condition:Trial	1.24	1	0.266
Condition:CorrectDoorLocation	1.27	1	0.260
Trial:CorrectDoorLocation	0.66	1	0.417
Condition:Trial:CorrectDoorLocation	1.01	1	0.314

Table S44. ANOVA of FreezingTime during training

	Chisq	Df	Pr(>Chisq)
Condition	0.42	1	0.519
Trial	10.58	1	0.001
CorrectDoorLocation	15.26	1	0.000
Condition:Trial	1.48	1	0.223
Condition:CorrectDoorLocation	3.42	1	0.064
Trial:CorrectDoorLocation	8.80	1	0.003
Condition:Trial:CorrectDoorLocation	0.81	1	0.367

Table S45. ANOVA of MovingTime during training

	Chisq	Df	Pr(>Chisq)
Condition	2.00	1	0.158
Trial	6.96	1	0.008
CorrectDoorLocation	0.07	1	0.796
Condition:Trial	1.13	1	0.288
Condition:CorrectDoorLocation	8.01	1	0.005
Trial:CorrectDoorLocation	0.65	1	0.422
Condition:Trial:CorrectDoorLocation	0.09	1	0.766

Table S46. ANOVA of AveHeading during training

	Chisq	Df	Pr(>Chisq)
Condition	3.27	1	0.071
Trial	0.01	1	0.928
CorrectDoorLocation	0.06	1	0.800
Condition:Trial	12.25	1	0.000
Condition:CorrectDoorLocation	4.53	1	0.033
Trial:CorrectDoorLocation	0.36	1	0.547
Condition:Trial:CorrectDoorLocation	0.00	1	0.967

Table S47. ANOVA of AveHeadingDoorAngle during training

	Chisq	Df	Pr(>Chisq)
Condition	6.44	1	0.011
Trial	0.86	1	0.353
CorrectDoorLocation	13.58	1	0.000
Condition:Trial	4.24	1	0.040
Condition:CorrectDoorLocation	7.50	1	0.006
Trial:CorrectDoorLocation	1.02	1	0.311
Condition:Trial:CorrectDoorLocation	1.23	1	0.268

Table S48. ANOVA of AveAngularSpeed during training

	Chisq	Df	Pr(>Chisq)
Condition	13.18	1	0.000
Trial	0.59	1	0.442
CorrectDoorLocation	11.47	1	0.001
Condition:Trial	6.42	1	0.011
Condition:CorrectDoorLocation	0.77	1	0.381
Trial:CorrectDoorLocation	0.66	1	0.417
Condition:Trial:CorrectDoorLocation	0.87	1	0.351

Table S49. ANOVA of AvePeakAngSpeed during training

	Chisq	Df	Pr(>Chisq)
Condition	1.14	1	0.285
Trial	1.86	1	0.172
CorrectDoorLocation	20.37	1	0.000
Condition:Trial	7.38	1	0.007
Condition:CorrectDoorLocation	2.45	1	0.117
Trial:CorrectDoorLocation	0.23	1	0.628
Condition:Trial:CorrectDoorLocation	0.12	1	0.724

Table S50. ANOVA of AveAcceleration during training

	Chisq	Df	Pr(>Chisq)
Condition	7.68	1	0.006
Trial	5.43	1	0.020
CorrectDoorLocation	1.49	1	0.222
Condition:Trial	0.35	1	0.557
Condition:CorrectDoorLocation	0.00	1	0.988
Trial:CorrectDoorLocation	0.47	1	0.492
Condition:Trial:CorrectDoorLocation	0.78	1	0.376

Table S51. ANOVA of AvePeakAcceler during training

	Chisq	Df	Pr(>Chisq)
Condition	2.07	1	0.151
Trial	0.58	1	0.447
CorrectDoorLocation	5.41	1	0.020
Condition:Trial	0.12	1	0.730
Condition:CorrectDoorLocation	3.57	1	0.059
Trial:CorrectDoorLocation	0.11	1	0.737
Condition:Trial:CorrectDoorLocation	0.00	1	0.979

Table S52. ANOVA of Heading during training

	Chisq	Df	Pr(>Chisq)
Condition	0.13	1	0.723
Trial	9.52	1	0.002
CorrectDoorLocation	59.87	1	0.000
Condition:Trial	3.19	1	0.074
Condition:CorrectDoorLocation	14.30	1	0.000
Trial:CorrectDoorLocation	1.18	1	0.278
Condition:Trial:CorrectDoorLocation	1.16	1	0.281

REFERENCES

Dominik, C. (2010). *The Org Mode 7 Reference Manual-Organize your life with GNU Emacs* (Network Theory Ltd.)

Schulte, E., Davison, D., Dye, T., Dominik, C., et al. (2012). A multi-language computing environment for literate programming and reproducible research. *Journal of Statistical Software* 46, 1–24
